# Genetic counseling and the role of genetic counselors in the United States

**DOI:** 10.1515/medgen-2021-2054

**Published:** 2021-05-14

**Authors:** Christian P. Schaaf

**Affiliations:** Heidelberg University, Institute of Human Genetics, Im Neuenheimer Feld 366, 69120Heidelberg, Germany; Baylor College of Medicine, Department of Molecular and Human Genetics, Houston, USA; Jan and Dan Duncan Neurological Research Institute, Texas Children’s Hospital, Houston, USA

**Keywords:** genetic counseling, genomic medicine, licensure

## Abstract

Genetic counselors represent an indispensable, well-established, and well-integrated group of healthcare providers in the field of genetic and genomic medicine in the United States. They work with other members of the healthcare team to provide information and support to individuals and families concerned with genetic disorders. With more than 5,000 certified genetic counselors in the U.S. and an expected growth of 100 % over the next decade, genetic counseling represents one of the fastest-growing professions in the U.S.

Genetic counselors work in clinical environments (e. g., hospitals), in companies (e. g., genetic testing firms), and as consultants to medical practices and others. Twenty-six states license genetic counselors as practitioners who can bill independently, with licensure applications underway in the remaining 24 states.

Physicians, genetic counselors, and diagnosticians represent the three pillars of comprehensive, integrated genomic medical care. Within this triad, genetic counselors see their primary role in procuring and interpreting family and medical histories, assessing inheritance, quantifying chances of recurrence, facilitating decision-making regarding genetic testing options, and explaining the results of such testing to the respective individuals within the greater context of their families.

## Introduction

The field of medical genetics has grown tremendously over the past decades, to the point that it now affects all medical specialties. The translation and implementation of genomic medicine into clinical care and the realization of the comprehensive clinical benefits of such implementation remain a key challenge for the healthcare providers involved [1]. The increasing application of diagnostic genetic testing in medicine leads to an increasing need for a “genomic workforce” to deliver care to the affected individuals and their families. This includes but is not limited to the adequate assessment of the most appropriate genetic tests, pre-test education, counseling and consenting, interpretation of diagnostic findings, and communication of these to the respective individuals and their families. New, collaborative interactions with specialists from various disciplines are required, and new training programs need to be developed in order to improve genomic healthcare and patient outcomes [2].

In Germany, genomic healthcare is currently delivered by physicians only. The German Genetic Diagnostics Act (GenDG) regulates that genetic testing for medical purposes may only be initiated and carried out by a physician [3]. While diagnostic testing may be conducted by any physician, predictive genetic testing may only be initiated by board-certified medical geneticists or physicians from other subspecialties, who have completed a certificate program for genetic counseling. The demand for genetic counseling has been steadily increasing and waiting times for appointments in genetics outpatient clinics have increased. At the same time, the number of key service providers, i. e., contracted medical specialists in human genetics, has remained more or less constant [4].

In the United States, the first class of Master’s degree genetic counselors graduated from Sarah Lawrence College in Bronxville, NY, in 1971. The new profession was meant to fill a gap between knowledge and service in the rapidly developing field of genetics. Genetic counselors were supposed to fill a niche at the fascinating intersection of laboratory, statistical, and psychological aspects of human genetics [5]. Since then, the majority of genetic services in the U.S. have been provided by genetic counselors and physicians (with admission of the American Board of Medical Genetics to the American Board of Medical Specialties as a primary specialty board in 1991). The continued progress in genetic service delivery has been attributed, to a large part, to the non-physician members of the genetics community, in particular the genetic counselors, operating in concert with the physicians [6].

In this article, I would like to review the role of genetic counselors in the U.S., discuss their interactions with physician providers as part of the genomics workforce, look at some challenges related to this cooperation, and assess the overall opportunities of interdisciplinary teams in genomic healthcare.

## Genetic counselors in the U.S.

As of 2019, there were more than 5,000 certified genetic counselors (American Board of Genetic Counseling), compared to approximately 1,700 physicians certified in clinical genetics (American Board of Medical Genetics and Genomics) in North America. Genetic counseling has been established as a graduate-level program since the late 1960s and is offered at more than 40 colleges and universities in the U.S. [7]. The Accreditation Council for Genetic Counseling (www.gceducation.org) establishes standards for accreditation, which are used in accrediting Master’s degree-granting programs that prepare individuals to enter the genetic counseling profession. All graduate programs in genetic counseling are required to provide training over a minimum of 21 months or two academic years. The required general content areas include but are not limited to the principles of human genetics and genomics, principles of genetic counseling and clinical genetics, psychosocial content, social/ethical/legal issues in genetics, healthcare delivery and principles of public health, education, research methods, and professional development. In addition to general training, fieldwork experiences must be part of genetic counselor training, with a minimum of 50 required participatory cases, of which at least 40 must be with individuals evaluated for risk of or affected by diverse genetic conditions across the lifespan.

Genetic counseling has been one of the most rapidly growing professions, with more than 100 % growth between 2010 and 2020. Of all genetic counselors in the U.S., 98 % have a Master’s degree in human genetics or genetic counseling. As a professional organization, the National Society of Genetic Counselors (NSGC) promotes the professional interests of genetic counselors and provides a network for professional communications (www.nsgc.org). The American Board of Genetic Counseling is in charge of certifications and recertifications of genetic counselors (www.abgc.net), and the Accreditation Council for Genetic Counseling accredits genetic counseling training programs (www.gceducation.org). The American Board of Genetic Counseling requires genetic counselors to be recertified every five years by reexamination or by completing continuing education courses (25 hours/year).

As per the 2020 professional status survey by the NSGC, over half of all genetic counselors in the U.S. have a direct patient care position (52 %), while 25 % have a non-direct patient care position and 23 % have a mixed position. The NSGC defines direct patient care as “a role that primarily involves counseling patients,” including case preparation, service delivery, and follow-up. Examples of non-direct patient care include laboratory involvement, teaching, marketing, and administrative roles [8]. More than 50 % of all genetic counselors work in clinical environments (hospitals, medical practices, etc.), while 17 % work for diagnostic laboratories and 11 % in the public service arena (health departments, etc.). Out of all genetic counselors in the U.S., 44 % work in cancer genetics (adult), 29 % in prenatal genetics, 25 % in pediatrics, 19 % in general adult genetics, and 21 % in preconception/reproductive genetics (with some working in more than one subspecialty, therefore total will add up to more than 100 %).

As of October 2020, there were 26 U.S. states issuing licenses for genetic counselors. The goal of licensure is to ensure that the licensees have the minimal degree of competency necessary to ensure that public health, safety, and welfare are protected. Without licensing, most genetic counselors cannot be credentialed by their employers and cannot bill for their services. Moreover, without licensure, genetic counselors are unable to practice independently, which means that they can only work under physician supervision. As of October 2020, in three U.S. states, bills had been passed for licensing of genetic counselors, and were in the process of rulemaking. In 18 states, the legislative process for licensing of genetic counselors was underway.

**Figure 1:**
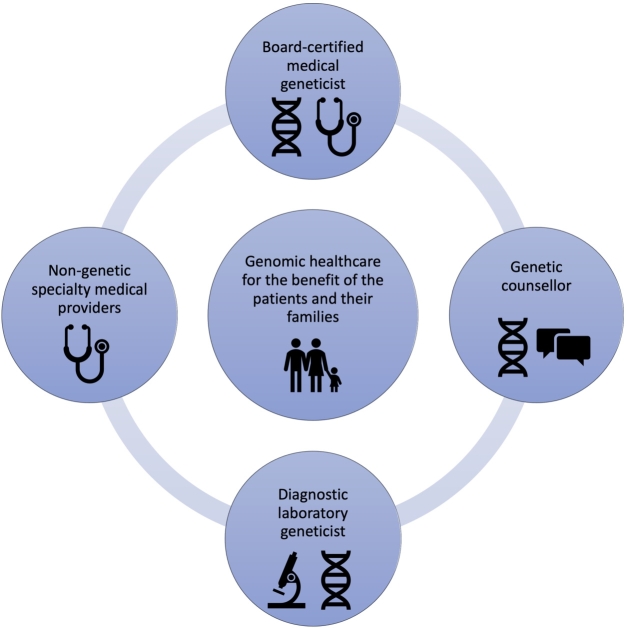
Medical geneticists, non-genetic medical providers, genetic counselors, and diagnostic laboratory geneticists work together to provide comprehensive, interdisciplinary healthcare for the benefit of their patients and their respective families.

## Areas of contention – billing and reimbursement

To this date, genetic counselors are not recognized as healthcare providers by the U.S. Centers for Medicare and Medicaid Services (CMS) and therefore, unlike nurse practitioners and physicians who can provide genetic counseling services, genetic counselors are not reimbursed under Medicare, a national health insurance program that provides health insurance for Americans aged 65 and older, as well as for younger individuals with disability status as determined by the Social Security Administration. A bill “Access to Genetic Counselor Services Act of 2019” (H.R. 3235) was introduced in the House of Representatives. This bill would authorize CMS to recognize certified genetic counselors as healthcare providers, allowing them to receive 85 % of what physicians receive for providing genetic counseling services to Medicare patients. When that bill was introduced in 2018, the then President of the American Society of Human Genetics, David Nelson, expressed the ASHG’s support for the legislation. However, the American College of Medical Genetics (ACMG) expressed concern about the bill, in particular the unrestricted, independent ordering of genetic tests as well as other diagnostic studies. The ACMG thereby supported the reimbursement of genetic counselors for genetic counseling services, but not for independent practice of medicine, highlighting the differences in medical training and expertise in comparison to genetic counseling. They emphasized that genetic tests that confer a diagnosis or guide treatment decisions require physician involvement. In addition, some genetic tests may require a medical evaluation, including physical examination and other types of laboratory testing to identify genetic testing needs. For these reasons, ACMG proposed minor amendments to H.R. 3235 to encourage the continuation of team-based models for patient care.

To this date, the ability of genetic counselors in the U.S. to bill for services independently still varies from state to state and is dependent on the insurance status of the patient(s) seeking genetic counseling services.

## Interdisciplinary genomic patient care

Interdisciplinary healthcare teams have become the new model for patient care delivery in today’s complex healthcare environment. This applies to all areas and disciplines, but in particular to individuals with complex rare diseases, resulting in a need for innovative healthcare structures and interdisciplinary working groups [9]. In a situation where not enough physician providers trained in medical genetics are available to deliver patient care, as is the case in Germany right now [4], innovative service delivery models may be required for the benefit of the patients and their families. As it relates to genetic and genomic healthcare, four main providers come to mind (Figure 1):

(1) **Physicians trained in medical genetics** (board-certified medical geneticists). They currently provide the majority of direct patient care in human genetics in Germany. They are fully trained in medicine in general, which comes with expertise in assessing patient symptoms, physical examination, interpretation of diagnostic findings, establishing a differential diagnosis, ordering the respective additional diagnostic tests, including genetic and genomic testing, communicating those findings with patients and their families, and taking the required steps for further treatment and management.
(2) **Non-genetic specialty medical providers**. They represent not only a major source of referrals to the genetics clinic, but also a critical liaison and partner to provide the necessary treatment and management after a genetic diagnosis is established. Given the growing availability of genetic testing and its implications in healthcare in general, more and more specialty-trained physicians in Germany decide to complete a certificate course for genetic counseling, which increases their proficiency in ordering genetic testing and allows them to initiate predictive genetic testing under German law.
(3) **Diagnostic laboratory geneticists**. They are either physicians or scientists with a different background (often biology), trained in establishing and executing genetic diagnostic testing. They can be formally trained and certified as “Fachhumangenetiker” by the German Society of Human Genetics, which can further be recognized as “European registered Clinical Laboratory Geneticist” by the European Board of Medical Genetics. Given the growing complexity and comprehensiveness of genetic and genomic testing, a close interaction between referring provider and laboratory geneticist becomes increasingly important. While the clinical indication for genetic testing of an individual patient should be determined by the physician caring the respective individual, the physician and the diagnostic laboratory geneticist may work together in determining the suitability of the requested test relative to clinical indication. This involves factors such as how well relevant genes are covered by next-generation sequencing technology, as well as which types of mutations may not be detected by the testing ordered (triplet repeats, copy number variants, non-coding variants, etc.) [1]. Even more importantly, laboratory geneticist and referring physician should interact when it comes to communicating the results of genomic testing, and when an integrated interpretation of those results is necessary. This involves the assessment of variants of uncertain significance, the interpretation of genetic variation in light of the specific clinical phenotype (“reverse phenotyping”), and the assessment of segregation or non-segregation within the family [10].
(4) **Genetic counselors** (currently not established an actual profession in Germany). They may work side-by-side with the medical geneticist or independently in patient care settings which require a lesser amount of actual medical expertise in a sense that would require training as a physician. The greatest strength of genetic counselors is often in the area of communication, both at the level of collecting information from the patient and in the area of communicating risk, delivering results of genetic testing to the patient, bringing that into the greater context of the patient’s family, and providing support and guidance based on the respective diagnosis.

**Figure 2:**
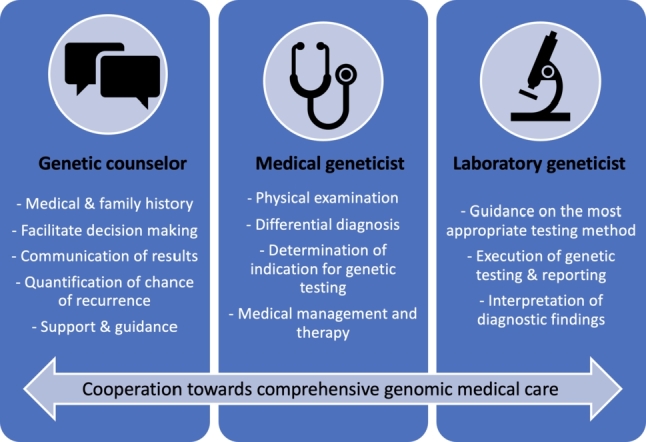
Various areas of expertise and specific skill sets come together and complement each other as part of comprehensive genomic medical care.

Figure 2 illustrates how specific expertise on the level of the genetic counselor, medical geneticist, and laboratory geneticist can complement each other and (when applied successfully in a team-based approach) lead to better, comprehensive genomic medical care, for the benefit of the individual patients and their respective families.

**Figure 3:**
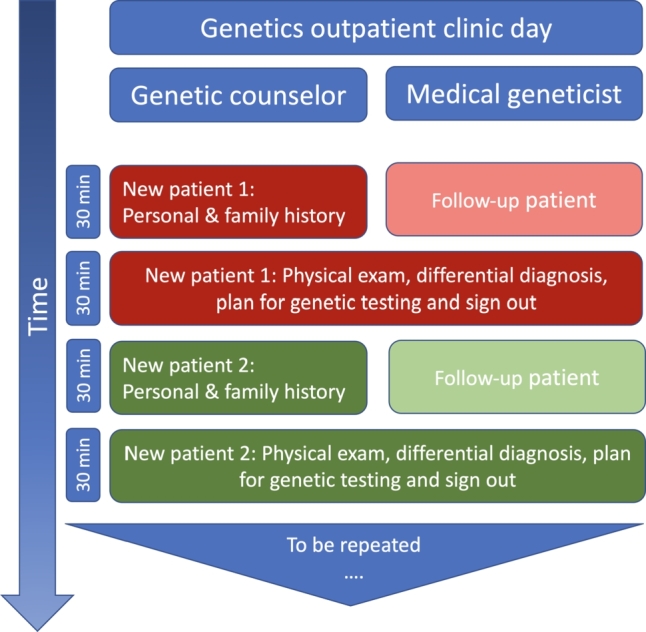
Example of an integrated clinic schedule, with a genetic counselor and medical geneticist seeing patients together. The genetic counselor starts with new patients, taking the personal medical and family history and reviewing prior genetic testing (approximately 30 minutes). At the same time, the medical geneticist sees a follow-up patient for discussion of genetic tests results, medical management, or other. After 30 minutes, the genetic counselor and medical geneticist meet to briefly discuss patient 1, then go into the exam room together, for the physician to perform the physical exam and to develop a differential diagnosis. Then genetic counselor and physician together with the family discuss genetic testing options and plans. After a total of 60 minutes, a new patient assessment and a follow-up patient assessment have been completed, and the next set of patients can be evaluated. Time frames are based on the personal experience of the author during his tenure at Texas Children’s Hospital (Houston, TX), with additional staff available to take vital signs, for rooming patients, and for phlebotomy services.

## Service delivery by genetic counselors/navigating the boundaries between physicians and non-physician healthcare providers

In the author’s opinion, it is critical to respect each other as full partners of a team rather than establishing a situation where one (the genetic counselor) provides the legwork for the other (the physician), when aiming to establish beneficial working relationships between medical geneticists and genetic counselors. Therefore, it is particularly important to recognize and consider the specific strengths of one another’s training and expertise (Figure 2). Consequently, an integration of genetic counselors into the genomic healthcare team will lead to a situation where they collect and integrate information, communicate results, provide counseling that answers individual patients’ questions, and offer support and guidance. Unique physician skill sets involve the interpretation of clinical symptoms, the comprehensive physical examination, the integration of such information into the establishment of a differential diagnosis, and the decision regarding further diagnostic workup. Once a diagnosis has been made and the genetic counselor has communicated findings to the patient, including risk for other family members, the physician’s role will be to integrate that diagnosis into a medical treatment and management plan and to involve other subspecialties, related to the specific diagnosis. In clinical practice, genetic counselors and physicians can work side-by-side as an actual genomic healthcare team, and their schedules can be intertwined in a way such that genetic counselors collect all necessary information (personal medical history and family history) and then step out to discuss the case with the physician, to then complete the assessment together with the physician, who will conduct the physical examination, establish the differential diagnosis, and develop a plan for (or against) genetic testing for the respective patient. Figure 3 outlines a clinic schedule, in which the physician sees follow-up patients while the genetic counselor collects information from new clinic patients. Alternatively, a physician could work with two genetic counselors, alternating between them to offer an integrated clinic schedule of only new patient visits. Importantly, the schedule and workflow shown here are based on personal experience of the author. As the skill sets of medical geneticists and genetic counselors overlap, the tasks assigned to each of the two professions may also overlap. The organization of actual workflows in an interdisciplinary clinical setting is therefore highly dependent on skill set, experience, and potentially even preference of each team member, but also subject to clinic scheduling, availability of additional medical personnel, and most importantly patient population.

In some settings, genetic counselors may work independently (if the legal situation allows). In the U.S., cancer genetics and prenatal genetics are subspecialties where genetic counselors have a particularly strong representation. This relates to the fact that these patient care settings require a lesser amount of evaluation that requires training as a physician, such as a detailed physical exam or generation of a complex differential diagnosis.

## Outlook and perspective

Germany appears to be at the brink of acknowledging genetic counselors as new profession, which will lead to an increase of genomic healthcare services, if applied successfully. The German Society of Human Genetics submitted a request to the Ministry of Health in 2019, asking for support in the implementation of “genetic counselor” as a new profession. The consequences of such would be far-reaching and may change the way genetic medicine is practiced in Germany. The exact role of genetic counselors, their integration within interdisciplinary teams, and their ability to bill for their services (or lack thereof) would require a broad discussion not only of the genetics community, but should also include law makers and health economists, as well as patient representatives.

Interestingly, at the same time in the U.S., where genetic counselors have been a stronghold of genomic healthcare for decades, a further expansion and integration of genomic healthcare is being discussed. Based on the rapid growth of genetic and genomic testing in medicine more generally, it has been highlighted that new educational mechanisms are required to reach providers both within and beyond the traditional genetic sectors, including nurses, physician assistants, and non-genetic physicians [2]. In addition, patients and their families need to be recognized as partners, as the paternalistic medicine has come to an end, and families are searching for information about rare diseases themselves and increasingly organize in rare disease groups, utilizing the tools of social media [11]. That being said, a great amount of flexibility and adaptability is necessary in order to adapt to the existing and expected need, and in order to maintain and further improve genomic healthcare. The future of genomic medicine is team-based, with patients and medical providers working hand-in-hand in a non-paternalistic way, genetic providers and providers from other subspecialties working together as a team, and medical geneticists embracing non-physician care providers as valuable contributors within their team.
